# Convergent Evolution of Adhesive Properties in Leaf Insect Eggs and Plant Seeds: Cross-Kingdom Bioinspiration

**DOI:** 10.3390/biomimetics7040173

**Published:** 2022-10-22

**Authors:** Thies H. Büscher, Stanislav N. Gorb

**Affiliations:** Department of Functional Morphology and Biomechanics, Institute of Zoology, Kiel University, Am Botanischen Garten 9, 24118 Kiel, Germany

**Keywords:** glue, Phylliidae, Cucurbitaceae, fiber reinforcement, biomimetics, ivy gourd

## Abstract

Plants and animals are often used as a source for inspiration in biomimetic engineering. However, stronger engagement of biologists is often required in the field of biomimetics. The actual strength of using biological systems as a source of inspiration for human problem solving does not lie in a perfect copy of a single system but in the extraction of core principles from similarly functioning systems that have convergently solved the same problem in their evolution. Adhesive systems are an example of such convergent traits that independently evolved in different organisms. We herein compare two analogous adhesive systems, one from plants seeds and one from insect eggs, to test their properties and functional principles for differences and similarities in order to evaluate the input that can be potentially used for biomimetics. Although strikingly similar, the eggs of the leaf insect *Phyllium philippinicum* and the seeds of the ivy gourd *Coccinia grandis* make use of different surface structures for the generation of adhesion. Both employ a water-soluble glue that is spread on the surface via reinforcing fibrous surface structures, but the morphology of these structures is different. In addition to microscopic analysis of the two adhesive systems, we mechanically measured the actual adhesion generated by both systems to quantitatively compare their functional differences on various standardized substrates. We found that seeds can generate much stronger adhesion in some cases but overall provided less reliable adherence in comparison to eggs. Furthermore, eggs performed better regarding repetitive attachment. The similarities of these systems, and their differences resulting from their different purposes and different structural/chemical features, can be informative for engineers working on technical adhesive systems.

## 1. Introduction

A core principle of biomimetics is to find inspiration for human problem solving in nature [1]. While several natural principles were successfully adapted in biomimetic studies in the past, sometimes a vague similarity to biological structures appears to be sufficient for some researchers to claim bioinspiration as a trademark to claim usefulness per se. However, such a top-down approach to back up technical innovations with supposed biological similarity does not necessarily use the full potential of biomimetic thinking [2]. Biological systems undoubtedly offer significant potential for inspiration for problem solving, as many functions in nature have evolved in response to specific environmental requirements and are subjected to continuous selection [3]. Several technical innovations are a result of investigation of examples from nature, for example, in the field of gripping devices in soft robotics [4,5,6]. However, the actual strength in investigating such phenomena lies in the understanding of the actual functional constraints these biological systems are adapted to and in disarticulation of the key functions. A great potential for finding inspiration in natural functional systems is especially present in systems that evolved convergently in different remotely related organisms. Within animals, one striking example of such a convergence is found in their adhesive systems [7]. As attachment, in general, is very important for many animals in different aspects of their life (nutrition, locomotion, dispersal, etc.) and various different taxa possess elaborate attachment systems [8,9]. These systems are widespread within the animal kingdom but occur in very different clades of animals and can have quite different morphology and functionality [7]. The functions of the different kinds of attachment devices themselves in turn are often tuned to more general requirements, as they rely on the physical constraints of the interaction of the attachment organ and the substrate. These constraints are universal for all species, independently of their relatedness, and, if the environment is similar, result in a similar morphology [10,11,12,13]. Consequently, a similar morphology can occur convergently as a result of the similar conditions the systems are adapted to in phylogenetically distinct lineages.

**Figure 1:**
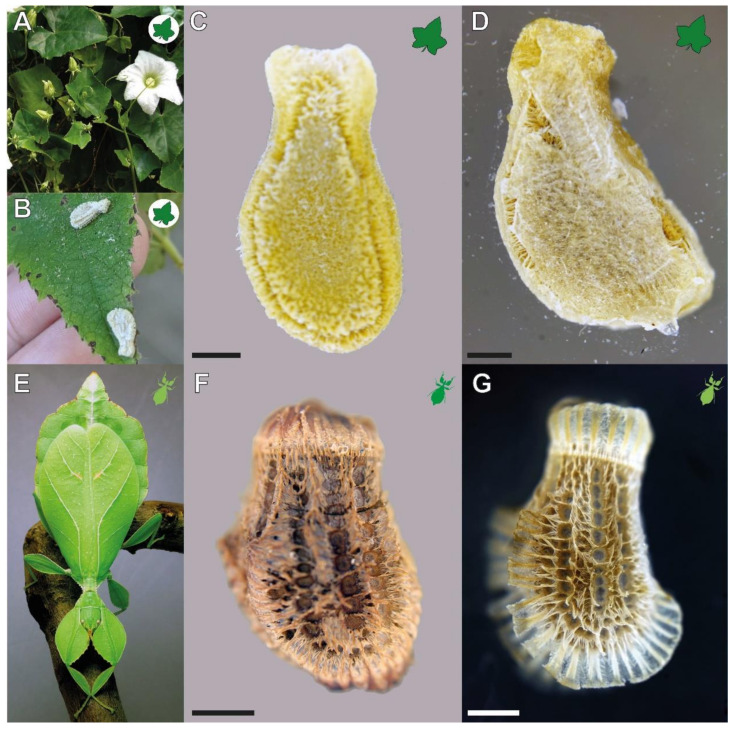
Focal organisms. (**A**–**D**) *Coccinia grandis*: (**A**) Flowering plant (modified from [16], published under CCBY 4.0). (**B**) Seeds found in the field attached to the leaves of a different plant (provided by Lih Wei-Song). (**C**) Extracted dry seed before water contact. (**D**) Seed attached to a glass slide, photographed through the slide. (**E**–**G**) *Phyllium philippinicum*: (**E**) Adult female (from [15] published under CCBY 4.0). (**F**) Dry egg before first water contact, lateral view. (**G**) Egg attached to a glass slide, photographed through the slide. Scale bars: 1 mm.

Such a mechanism with a similar functionality in two organisms from phylogenetically distant lineages is represented by the adhesive mechanism of the eggs of the Philippine leaf insect *Phyllium philippinicum* Hennemann, Conle, Gottardo & Bresseel, 2009 (Phasmatodea, Phylliidae) and the seeds of the ivy gourd *Coccinia grandis* (L.) Voigt (Cucurbitaceae). Both systems include fibrillary surface structures on the surface and a glue component that is applied to the substrate. While the adhesive mechanism of leaf insect eggs has already been investigated [14,15], the adhesiveness of *C. grandis* seeds has, so far, no apparent notion in the literature. However, the seeds of this plant carry a similar adhesive system to that described for the eggs of walking leaf insects. Furthermore, they are so similar in appearance that they are easily confused with each other in the field because both species co-occur in similar environments. We became aware of this species and the similarity of both reproductive stages due to this confusion. During a field trip in Pasir Ris, Singapore (1°23′33.2″ N 103°55′33.6″ E), the supposed eggs of a Singaporean *Phyllium* sp. were found adhered to local plants (Figure 1B). However, closer inspection and incubation of these eggs revealed their true identity as C. *grandis* seeds (pers. comm. Wei-Song Lih).

**Figure 2:**
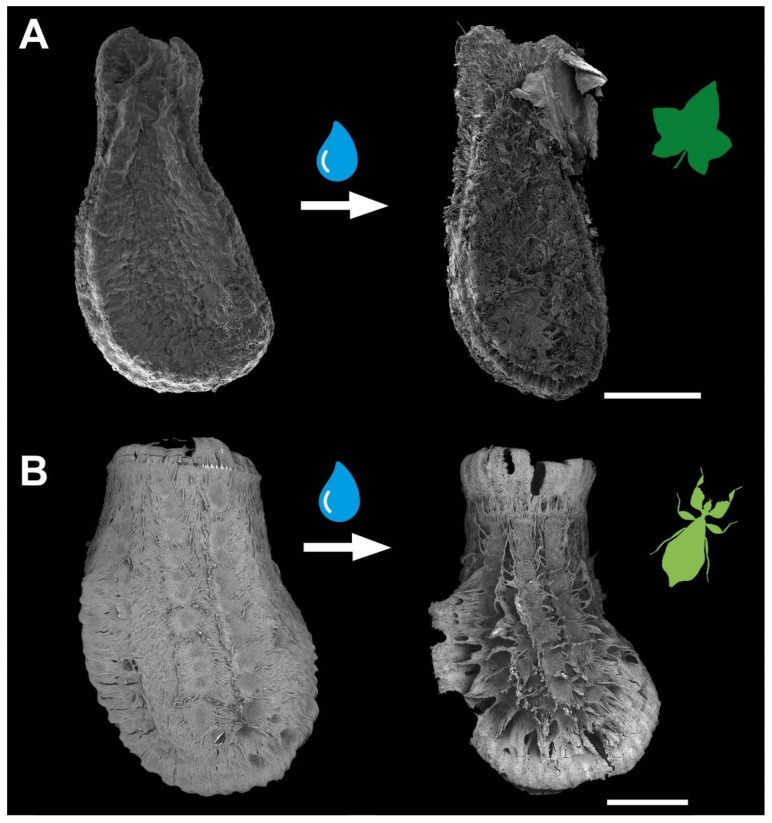
Water interactions of both adhesive systems. (**A**) Seed of *Coccinia grandis*. (**B**) Egg of *Phyllium philippinicum* (modified from [15] published under CCBY 4.0). The surface structures of both organisms are densely packed. Water contact induces the spreading of fibrillary adhesive structures, which carry glue for triggering adhesion in contact with the substrate. Scale bars: 1 mm.

As described for *P. philippinicum* eggs [14,15], these seeds display adhesive capability after activation with water (Figure 2). Likewise, they carry fibrillary adhesive structures on their surface, which expand after exposure to water and adapt to the geometry of the substrate’s surface. Adhesion is also facilitated by a film of glue, which is distributed on the substrate. Details of this mechanism have been experimentally tested for leaf insect eggs, yielding a study on the influence of the roughness and surface chemistry [15] and the influence of different solvents on the activation of the exochorionic structures involved in it [14]. We aimed to investigate the mechanism of the seed adhesion of *C. grandis* in similar detail. This included an investigation of the morphology of the components involved in the adhesive mechanism and experimental characterization of its function under different substrate constraints. Both were used to compare the morphology and function of the two similar adhesive systems found in different kingdoms of life. The similarities and differences between the two species can be used to evaluate the common characteristics that are important for this kind of adhesive system and the modifications for specific tasks. Furthermore, it provides insights into the specific mechanisms in light of their ecological role, which might facilitate or prevent dispersal.

Natural adhesive systems can be tuned to fulfill specific tasks, such as coping with the crystalline wax coverage of plants [17], with strong torrents in fast flowing water [18,19], or the challenging surface and motion of the host in the case of parasites [20]. Others cope with a variety of influences at once [7,8,9,21,22]. Comparative approaches, such as the one presented herein, can help to understand the main principles of these systems and provide insights into the essence of the common principle. Such knowledge is useful for biomechanics to isolate the key characteristics of natural systems and evaluate the adaptations for the actual tasks of the respective examples. Consequently, the comparative investigation of such two similar mechanisms yields an evaluation of the biomimetic potential of the underlying common principle. To test the similarity of two similar natural adhesive systems from two different kingdoms, namely plants and animals, we herein characterized the adhesive system of *C. grandis* seeds in light of the present knowledge of the adhesive system of *P. philippinicum* eggs and compared the two systems in terms of their morphology, the adhesive performance on varying surface roughness and surface chemistry, and the repeatability of adhesion, which has already been shown for the leaf insect eggs [15]. To provide a similar base of knowledge of *C. grandis* seeds, we conducted the same analysis of the morphology using light and scanning electron microscopy and mechanically tested the resulting adhesion with the same setup used for *P. philippinicum* eggs in previous studies.

We specifically investigated the following questions:

1. How does the morphology differ between *P. philippinicum* eggs and *C. grandis* seeds?
2. What influence does the substrate surface roughness have on the adhesion of *C. grandis* seeds?
3. What influence does the surface chemistry have on the adhesion of these seeds?
4. What are the similarities and differences between both examples in terms of their adhesive performance and the repeatability of adhesion?

The results are discussed in the background of their significance for biomimetics.

## 2. Materials and Methods

### 2.1. Specimens

This study explored two different focal objects: The seeds of the ivy gourd *Coccinia grandis* (L.) Voigt (Cucurbitaceae) and the eggs of the Philippine leaf insect *Phyllium philippinicum* Hennemann et al., 2009 (Phasmatodea, Phylliidae). Measurements of *P. philippinicum* eggs were used for comparison of the two mechanisms and were previously published in Büscher et al. [15]. Novel data for *C. grandis* were obtained using the same methodology used therein to warrant comparability. Eggs were obtained directly after oviposition from female insects from a captive breeding culture. *C. grandis* seeds were obtained from Danushka Hiruni (Kudaweda, Sri Lanka). They were harvested by mechanical extraction, dried, and stored in a dry environment until experimental use. Both the seeds and eggs were weighed with an AG204 Delta Range microbalance (Mettler Toledo, Greifensee, Switzerland; d = 0.1 mg).

### 2.2. Microscopic Visualization

Both the seeds and eggs were imaged prior to attachment using a microscope (M205, Leica Microsystems Ltd., Wetzlar, Germany). Furthermore, they were photographed while attached to microscopy glass slides from two directions (above and below the glass). Images were taken using the microscope camera Leica DFC420 (Leica Microsystems Ltd., Wetzlar, Germany). We recorded stacked multifocus images and merged them using the software Leica Application Suite (LAS) version 3.8.0 (Leica Microsystems Ltd., Wetzlar, Germany).

Further, the eggs and seeds were examined with the SEM Hitachi TM3000 (Hitachi High-technologies Corp., Tokyo, Japan) at an acceleration voltage of 10 kV to obtain overviews and with the SEM Hitachi S4800 (Hitachi High-technologies Corp., Tokyo, Japan) at an acceleration voltage of 5 kV to obtain the morphological details. Images were processed in Photoshop CS6 (Adobe Systems Inc., San Jose, CA, USA). The samples were either prepared in the untreated condition (before contact with water) or after contact with water and the corresponding attachment to the glass. Both untreated and detached samples were air-dried and sputtered with 10 nm gold-palladium.

### 2.3. Detachment Force Measurements

**Figure 3:**
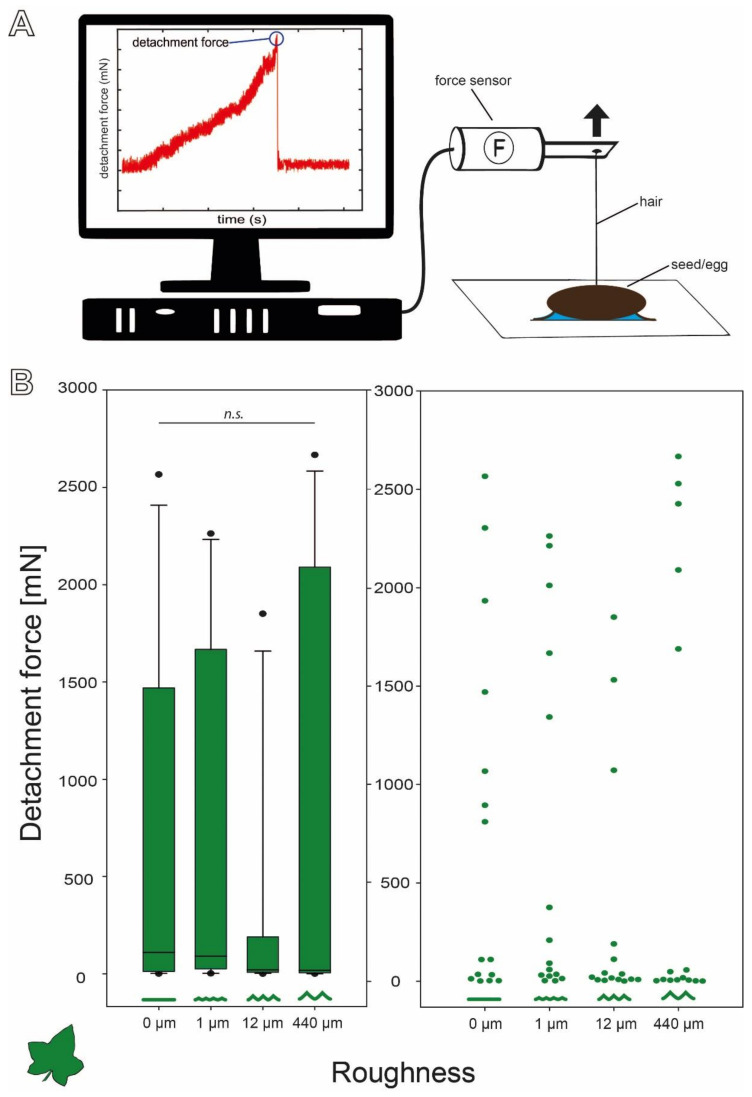
Detachment force measurements. (**A**) Schematic of the experimental setup with an example force–time curve of the detachment force measurement. (**B**) Detachment forces of *C. grandis* seeds on substrates with different roughness (*N* = 15 per substrate) represented by box plots (left) and jitter plots (right). Boxes indicate the 25th and 75th percentiles, the line represents the median, and the whiskers are the 10th and 90th percentiles. ***n.s***. = no statistical difference *p* = 0.55; Kruskal–Wallis ANOVA on ranks).

To compare the properties of both adhesive systems of eggs and seeds, the detachment forces of individual *C. grandis* seeds were measured with the same setup used in [15] for leaf insect eggs. The respective experimental samples were mounted on the standardized surfaces by individually placing them in droplets of distilled water (~100 μL) to activate the adhesive system. Afterwards, they were allowed to dry for 24–48 h and then individually connected to a force transducer (FORT1000, World Precision Instruments Inc., Sarasota, FL, USA) using bees wax to glue a horsehair onto the exposed side of the sample (Figure 3A). A BIOPAC Model MP100 and a BIOPAC TCI-102 system (BIOPAC Systems, Inc., Goleta, CA, USA) were used to record the detachment force–time curves of the samples from the substrates using the software Acqknowledge 3.7.0 (BIOPAC Systems Inc., Goleta, CA, USA). This was achieved by manually lowering the experimental substrates orthogonal to the sensor with a laboratory lifting platform at a speed of 2–3 cm/s. The maximum detachment force was determined by selecting the highest peak of the force–time curve. The detachment forces were measured in three set-ups:

1. Surface roughness

Four surfaces made of epoxy resin with different roughness were used as substrates for attachment of the samples (0, 1, 12, and 440 µm). For each substrate, 15 individual seeds and 32 individual eggs [15] were used.

2. Surface chemistry

Three surfaces with different surface free energy were used as substrates for the attachment of the samples. The surfaces had different water contact angles: 36.25 ± 1.15° (mean ± SD, *n* = 10) (hydrophilic), 83.38 ± 0.89° (hydrophobic), and 98.9 ± 0.47° (hydrophobic). For each substrate, 15 individual seeds and 20 individual eggs [15] were used.

3. Cyclic repetitions of attachment

The samples were subjected to repetitive individual pull-off measurements. After detachment, the same individual sample was reattached with a droplet of water. This procedure was repeated 6× for the eggs and 3× for the seeds. Furthermore, eggs were repetitively measured on the 0 µm epoxide substrate while the seeds were measured on all four different epoxide roughness test substrates. For each substrate, 15 individual seeds were used and 8 individual eggs [15] were used for the smooth substrate. If a sample did not adhere to the substrate at all, the detachment force of the individual sample was considered 0 mN, but the same individual was used again for subsequent measurements.

All measurements were carried out at a 20–23 °C temperature and 45.0–47.6% relative humidity. Except for the cyclic repetition experiments, neither the seeds nor eggs were used for more than one detachment force measurement.

### 2.4. Substrate Preparation

We used two different types of substrates: Epoxy resin with a range of surface roughness and glass with different wettability.

#### 2.4.1. Glass

Microscope objective glass slides (Carl Roth GmbH & Co. KG, Karlsruhe, Germany) were cleaned with isopropylic alcohol and used untreated as the hydrophilic substrate. Clean glass sides were silanized following Voigt and Gorb [17] to reduce the surface free energy and used as a hydrophobic substrate. The wettability was quantified by measuring the water contact angle of the substrates (aqua Millipore, droplet size = 1 μL, sessile drop method; *n* = 10 per substrate) with an OCAH 200 (Dataphysics Instruments GmbH, Filderstadt, Germany). The contact angle of water was 36.25 ± 1.15° on untreated glass and 98.9 ± 0.47° on silanized glass.

#### 2.4.2. Epoxy Resin

We used epoxy resin [23] and the two-step molding protocol of Salerno et al. [24] to obtain test substrates with different roughness. Glass with a 0 µm roughness, fine polishing papers (standardized roughness 1, 12 μm; Buehler, Lake Bluff, IL, USA), and industrial polishing paper with a 440 µm particle size were templates for the-two step molding. Negatives were created with polyvinylsiloxane-based dental wax (Colthéne/Whaledent AG, Altstatten, Switzerland) and filled with epoxy resin, which was cured at 70 °C for 24 h. The water contact angle of the epoxy resin was 83.38 ± 0.89° (mean ± SD, *n* = 10) [15].

### 2.5. Statistical Analysis

Statistical analyses were performed with SigmaPlot 12.0 (Systat Software Inc., San José, CA, USA). First, the data was tested for a normal distribution (Shapiro–Wilk test) and equal variance (Levene’s test). Due to the non-normality or missing homoscedasticity in all comparisons, only non-parametric tests were chosen. The detachment forces of the seeds on varying substrate roughness and on substrates with different surface chemistry were compared with Kruskal–Wallis one-way analyses of variance (ANOVA) on ranks and Tukey’s post hoc test. The novel data of the *C. grandis* seeds were compared with the previously reported data of *P. philippinicum* eggs [15] for these two scenarios for each substrate using the Mann–Whitney rank sum test. The repetitive measurements of both the seeds and the eggs were compared for each substrate with Friedman’s repeated measures ANOVAs and Tukey’s post hoc test. 

## 3. Results

### 3.1. Morphology

Both the eggs and seeds are laterally flat and remarkably similar in their overall appearance despite the different affiliations of the two species within distinct kingdoms. Hence, since detailed descriptions of the morphology of both reproductive structures (eggs and seeds) can be found in [15] for *P. philippinicum* eggs and [25] for *C. grandis* seeds, we only focused on the functionally relevant features.

**Figure 4:**
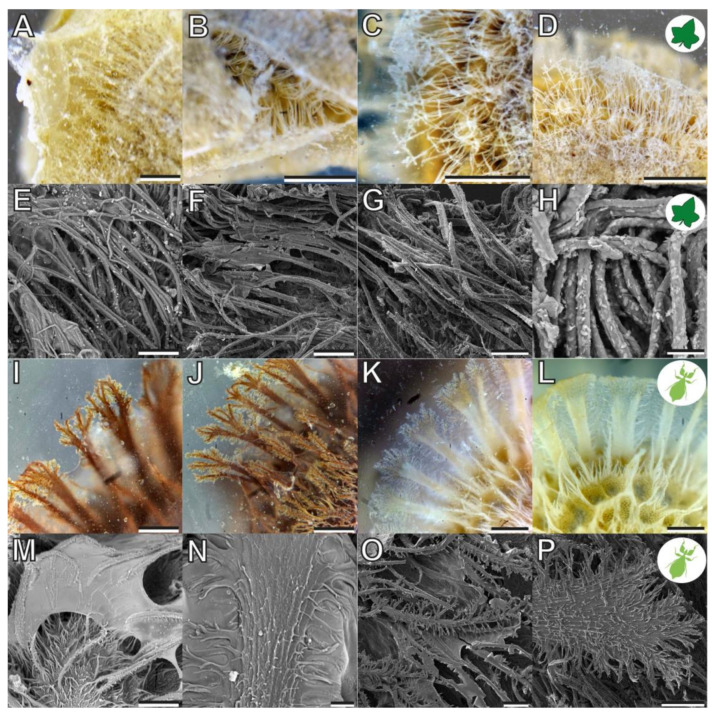
Morphology of the **fas** in the corresponding reproductive stages of the focal species. (**A**–**H**) Coccinia grandis seed. (**I**–**P**) Phyllium philippinicum egg. (**A**–**D**,**I**–**L**) Photographs of glue and **fas** interactions. (**E**–**H**,**M**–**P**) SEM of the glue and the **fas** morphology. Scale bars: (**A**–**D**,**I**–**L**) 300, (**E**–**G**,**N**,**O**) 50, (**H**) 20, (**M**) 150, and (**P**) 100 µm.

Both reproductive structures respond to water by expanding their fibrillar adhesive structures (**fas**). Prior to water contact, the *C. grandis* seed has a smooth surface due to the presence of a membrane (Figure 2A) under which the **fas** are tightly packed on the surface of the seed (Figure 4A,H). The **fas** of *C. grandis* seed are elongated, undivided filaments, which are mantled with a film of a hardened glue (Figure 4E,F). The length of the filaments is rather homogeneous and approximately 500 µm for the majority of them (see also [25]). Upon contact with water, the **fas** fan out and extend towards the substrate (Figure 4B–D). The tips of the **fas** make contact with the substrate and form a dense layer, adapting to the surface profile (Figure 4B). The glue is unevenly distributed along the length of the **fas** and accumulates on their tips, forming a continuous layer in combination with the **fas** (Figure 4B,C).

The **fas** of *P. philippinicum* also lie on the surface of the egg in the dry state, similar to the seeds (Figure 1F). However, both the distribution and the shape of the **fas** differ from the **fas** of *C. grandis* seeds. The so-called pinnae of these eggs are not homogenous in shape but consist of a central branch, which hierarchically splits into many finer terminal filaments (Figure 4I–L,N–P). Furthermore, they are oriented to the lateral rims of the egg and form two main rows and a collar at the operculum of the egg (Figure 1F,G). Smaller pinnae are present on the rest of the surface. The larger **fas** of these eggs are larger than the **fas** of the seeds but smaller ones are also present with fibrilllar structures in the heterogenous size range on the exochorion. Similar to the situation in seeds, a glue is present here, which mantles the **fas** in the dry state and spreads onto the substrate after water contact (Figure 4M–O). The **fas** respond to water by a similar expansion, forming a less continuous layer with the **fas** themselves in comparison to the one of seeds. The glue builds a closed film on the substrate in both organisms (Figure 4I–K). The tips of the pinnae carry less glue; instead, the glue is kept in the proximal space closer to the egg itself and is trapped between the **fas** and the egg (Figure 4I,J). 

### 3.2. Adhesion of C. grandis Seeds

#### 3.2.1. Influence of Substrate Roughness

The detachment forces of *C. grandis* seeds revealed a wide range of forces (Figure 3B). All four sets of measurements included particularly high detachment forces, but the overall distribution of the forces was strongly left skewed with much lower median detachment forces on all four substrates. However, the median detachment forces decreased with increasing substrate roughness. The median (±s.d.) detachment forces were 110.48 (±887.71) mN on 0 µm roughness, 91.23 (±883.72) mN on 1 µm, 20.37 (±598.02) mN on 12 µm, and 17.61 (±1086.81) mN on 440 µm. Nevertheless, several individual seeds detached at much higher pulling forces ranging up to 2600 mN on all four substrates (Figure 3B). This strong variation resulted in no significant differences between the detachment forces on the four substrates despite decreasing medians (Kruskal–Wallis one-way ANOVA on Ranks, H = 2.089, d.f. = 3, *p* = 0.554, *N* = 15 per roughness).

#### 3.2.2. Influence of Surface Chemistry

**Figure 5:**
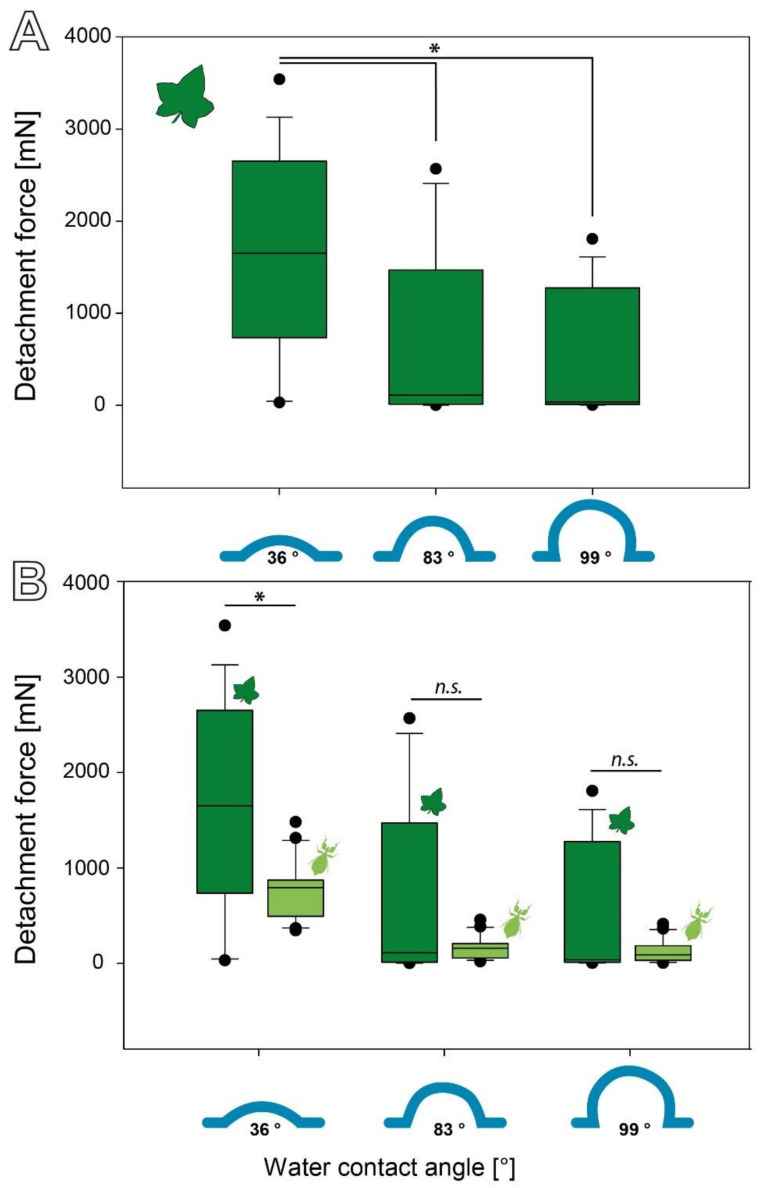
Influence of the surface wettability on the detachment forces. (**A**) Detachment force of Coccinia grandis seeds on substrates with different surface chemistry (*N* = 15 per substrate) * *p* ≤ 0.05 (Kruskal–Wallis one-way ANOVA on Ranks). (**B**) Comparison of the detachment forces of seeds and eggs (N_seeds_ = 15; N_eggs_ = 20). ***n.s.*** = no statistical difference; * *p* ≥ 0.05; Mann–Whitney rank sum test). Boxes indicate the 25th and 75th percentiles, the line represents the median, and the whiskers are the 10th and 90th percentiles.

The *C. grandis* seeds attached strongly to the hydrophilic substrate (Figure 5A). While the medium detachment force of the seeds on the substrate with a water contact angle of 36° wzs 1651.78 (±1083.55) mN, it was significantly lower (Kruskal–Wallis one-way ANOVA on Ranks, H = 10.992, d.f. = 2, *p* = 0.005, *N* = 15 per substrate; Tukey’s test *p* < 0.05) on the substrates with a contact angle of 83° (110.48 (±887.71) mN) and 99° (37.16 (±668.30) mN). The forces did not differ statistically between the substrates with contact angles of 83° and 99° (Tukey’s test *p* > 0.05).

#### 3.2.3. Cyclic Repetition

**Figure 6:**
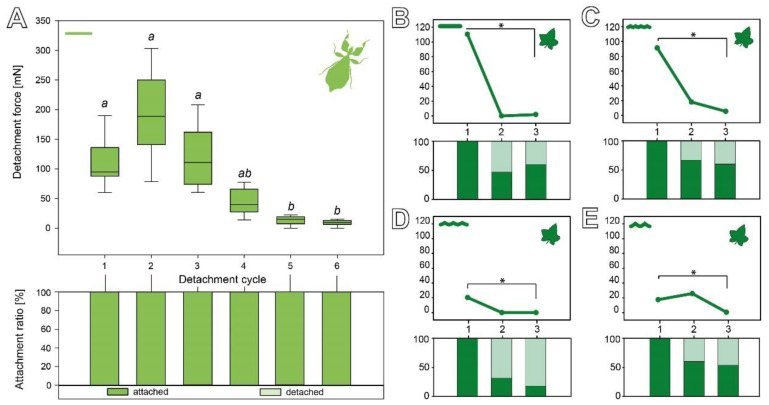
Sequential detachment force measurements. (**A**) *P. philippinicum* eggs. Line plot (above) and the corresponding count of attached and detached eggs (attachment ratio, below) for sequential repetitions (*N* = 8). Boxes indicate the 25th and 75th percentiles, the line represents the median, and the whiskers are the 10th and 90th percentiles. Lowercase letters indicate statistical similarity: boxes with the same letters are not statistically different (Friedman repeated-measures ANOVA on ranks, Tukey’s post hoc test, *p* < 0.05). (**B**–**E**) Detachment force of *C. grandis* seeds during repetitive detachments on different substrates (*N* = 15 per substrate). Line plots (above) and the corresponding attachment ratio (below) for sequential measurements; dots represent the median. (**B**) 0 µm. (**C**) 1 µm. (**D**) 12 µm. (**E**) 440 µm. * *p* ≤ 0.05 (Friedman repeated-measures ANOVA on ranks or repeated-measures ANOVA, respectively; Tukey’s post hoc test, *p* < 0.05).

The sequence of the detachment repetitions of *C. grandis* seeds on the four substrates with different roughness is shown in Figure 6B–E. The detachment forces significantly decreased on all four substrates from the first to the third cycle. While all individual seeds adhered in the first cycle, different amounts of seeds did not adhere in the second and third cycle depending on the substrate they were measured on. While the initial detachment force differed depending on the substrate (see Section 3.2.1), the subsequent cycles all showed reduced detachment forces (Figure 6B–E). On the smooth substrate, the median detachment force significantly decreased from 110.48 (±887.71) mN in the first cycle to 1.86 (±304.59) mN in the third cycle (Friedman repeated-measures ANOVA on ranks, χ^2^ = 8.98, d.f. = 2, *p* = 0.011; Tukey’s test *p* < 0.05). While 100% of the seeds adhered in the first cycle, only 47% adhered in the second cycle and 60% in the third cycle (Figure 6B). A substrate roughness of 1 µm resulted in the median detachment forces significantly decreasing from 91.23 (±883.72) mN in the first cycle to 5.50 (±220.87) mN in the third cycle (repeated-measures ANOVA, F = 3.51, d.f. = 2, *p* = 0.044; Tukey’s test *p* < 0.05). Compared to the other substrates, a higher fraction of seeds adhered in the later cycles: 100% in the first cycle, 67% in the second cycle, and 60% in the third cycle (Figure 6C). Measurements on the 12 µm rough substrate revealed a significant decrease in the detachment force from 20.37 (±598.02) mN in the first cycle to 0.00 (±62.24) mN in the third cycle (Friedman repeated-measures ANOVA on ranks, χ^2^ = 10.308, d.f. = 2, *p* = 0.006; Tukey’s test *p* < 0.05). The 12 µm roughness had the strongest impact on the attachment ratio: 100% of the seeds adhered in the first cycle, only 33% adhered in the second cycle, and 20% in the third cycle (Figure 6D). On the roughest substrate, the eggs showed a significant decrease in the median detachment forces from 17.61 (±1086.67) mN in the first cycle to 0.63 (±184.67) mN in the third cycle (Friedman repeated-measures ANOVA on ranks, χ^2^ = 9.927, d.f. = 2, *p* = 0.007; Tukey’s test *p* < 0.05). In the subsequent repetitions, they adhered more reliably, again with 100% of the seeds adhering in the first cycle, 60% in the second cycle, and 53% in the third cycle (Figure 6E).

### 3.3. Comparison of the Attachment Capability between Eggs and Seeds

**Figure 7:**
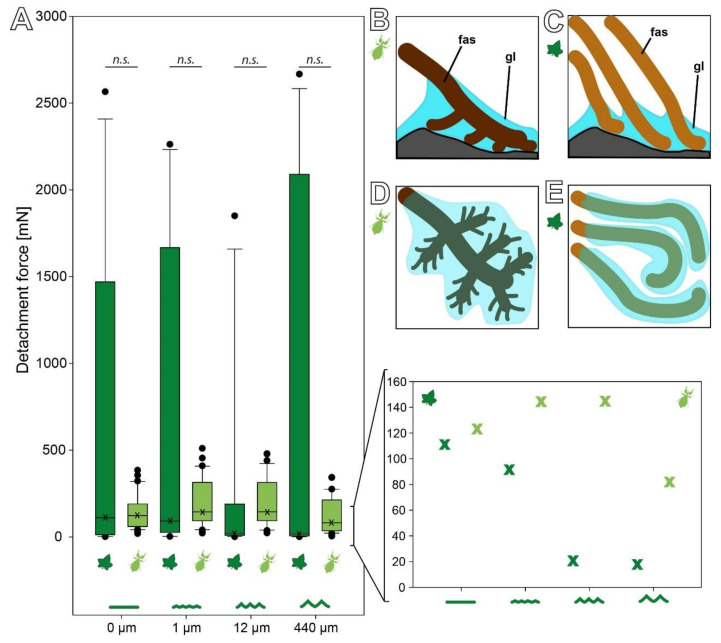
Comparison of the detachment forces of seeds and eggs on substrates with different substrate roughness. (**A**) Detachment forces are represented by box plots. Boxes indicate the 25th and 75th percentiles, the line represents the median, and the whiskers are the 10th and 90th percentiles. ***n.s***. = no statistical difference *p* > 0.05; Mann–Whitney rank sum test). Eggs (*N* = 32) are shown in light green, seeds (*N* = 15) in dark green. The enlargement shows the median of the respective detachment forces for clearer representation. X = median. (**B**–**E**) Schemes of the contact formation with rough substrates (**B**,**C**) and the glue distribution (**D**,**E**) of the expansions of eggs (**B**,**D**) and seeds (**C**,**E**). **fas** = fibrillary adhesive structure; gl = glue.

The detachment forces on substrates with different roughness show a similar pattern of substrate dependence for both the eggs and seeds (Figure 7A). Both reproductive stages revealed no statistically significant difference in regard to the roughness within the respective species (see Section 3.2, [15]); however, comparison of the medians of each experimental group yields a different behavior between the two adhesive systems. While the median detachment force of the *C. grandis* seeds strictly decreases with an increasing roughness, the median detachment forces of the *P. philippinicum* eggs are higher on the 1 and 12 µm rough substrates than on the smooth and rougher ones (Figure 7A). Nevertheless, due to the strong variation of the seeds, there was no statistically significant difference between the eggs and seeds on any of the four substrates. There was no significant difference between both reproductive stages on 0 (Mann–Whitney rank sum test, U = 179.00, T = 421.00, N_eggs_ = 32, N_seeds_ = 15, *p* = 0.167), 1 (Mann–Whitney rank sum test, U = 214.00, T = 334.00, N_eggs_ = 32, N_seeds_ = 15, *p* = 0.561), 12 (Mann–Whitney rank sum test, U = 179.00, T = 299.00, N_eggs_ = 32, N_seeds_ = 15, *p* = 0.167), and 440 µm (Mann–Whitney rank sum test, U = 219.00, T = 334.00, N_eggs_ = 32, N_seeds_ = 15, *p* = 0.914).

The surface chemistry affected the detachment forces in both species. Both showed significantly decreasing detachment forces with an increasing water contact angle of the substrate (Figure 5, [15]). Hydrophilic substrates (water contact angle of 36°) caused the highest detachment forces in both cases, but the seeds adhered significantly stronger to this substrate than the eggs (Mann–Whitney rank sum test, U = 81.00, T = 339.00, N_eggs_ = 20, N_seeds_ = 15, *p* = 0.022). The hydrophobicity of the substrate resulted in lower detachment forces but no significant difference between the eggs and seeds for an 83° (Mann–Whitney rank sum test, U = 142.00, T = 278.00, N_eggs_ = 20, N_seeds_ = 15, *p* = 0.803) and 99° water contact angle (Mann–Whitney rank sum test, U = 140.00, T = 260.00, N_eggs_ = 20, N_seeds_ = 15, *p* = 0.751).

During repetitive detachment events, eggs and seeds performed differently on two main aspects (Figure 6). The eggs of *P. philippinicum* retain repeatable attachment capability over some cycles. Although the detachment forces were significantly lower in the fifth and sixth cycle compared to the first three cycles (Friedman repeated-measures ANOVA on ranks, χ^2^ = 35.358, d.f. = 5, *p* ≤ 0.001; Tukey’s test, *p* < 0.05), all of the eggs adhered rather strongly and none failed to make sufficient contact during the attachment process. The seeds, in contrast, showed a fast decay of the detachment force starting from the second cycle and a high failure rate during the attachment repetitions, especially on the substrate with a 12 µm roughness (Figure 6B–E).

## 4. Discussion

The overall appearance and the general adhesive mechanism are similar in the reproductive stages of the two species examined here. However, the details of the specific adhesive performance differ between the two species and result in different advantages and disadvantages for both. While the offspring of the leaf insect *P. philippinicum* relies on the presence of suitable foodplants and should be protected during embryonic development [26,27], *C. grandis* seeds need to come into contact with soil for germination and to put down roots [26]. Consequently, the actual role of the adhesiveness of the two species differs, which is reflected in the differences in both the morphology and the resulting functionality.

### 4.1. Comparison between Seed and Egg Adhesive Systems

#### 4.1.1. Morphology

In addition to the similar overall appearance, *C. grandis* seeds and *P. philippinicum* eggs share several morphological characteristics. These include (1) the presence of fibrillary adhesive structures (**fas**) on the outer surface, (2) the presence of glue, and (3) the response to water of both components (Figure 2). Naturally, as one object is a plant and the other is an animal, these shared features are not homologous to each other. The specific shape of the **fas** differs in detail (Figure 4), which leads to some differences in their functionality. Obviously, the **fas** of both species are formed by completely different structures of different chemical and developmental origins. 

The eggs of phasmids are distinct from those of most other insect orders. The main specialty is the strong, hardened outer shell [28]. The egg capsule consists of two layers: the endochorion and the exochorion, which are both multi-layered [29,30]. The exochorion consists of different layers, which are structurally and chemically different, and is a product of the follicle cells [31]. Most noteworthy are the thick layer of calcium carbonate and the layer of calcium oxalate, with both being particularly tough and unique among insects [30,32]. The apomorphic toughening of the outer chorion enabled modifications for a plethora of different functions on the surface, for example, the adhesive system of the pinnae of *P. philippinicum*. The pinnae are formed on the outer surface of the egg capsule as secondary (follicular) secretions [31]. They are of variable length and width and hierarchically split several times.

The seeds of *C. grandis*, in contrast, carry more uniform **fas**. These are slender and of a rather uniform length (~500 µm). The outer layer (testa) of this seed generally consists of four layers, of which the outermost is an epidermal layer [25]. The epidermis of the closely related *Cocconia abyssinica* (Lam.) Cogn. is, according to Holstein [25], disintegrated and the cell walls of the epidermal cells form 500-µm-long **fas**. Other sources interpret the fibrils of the outer surface of *Coccinia* seeds of other species to be a fibrillose testa [33,34], or a disintegrated exotesta in *C. grandis* in particular [35]. It is likely that the **fas** of all *Coccinia* seeds with such a fibrillary surface are formed by the epidermis. In comparison, the **fas** of *C. grandis* seeds are more uniform and slender while the **fas** of *P. philippinicum* eggs are of a variable length and thickness and show hierarchically branching. 

There is, to our knowledge, no notion of the glue of *C. grandis* seeds (Figure 4B–D) in the literature. However, the seeds are encapsuled in a hyaline juicy envelope within the fruit, which seems to originate from carpellary tissue [36], which might partially remain on the seed surface and develop adhesive properties on the **fas**. Other plant seeds produce mucilage envelopes on their surface [37,38], which can be either pectin [39], hemicellulose, or cellulose dominated [40] and facilitate adhesion of the seeds. The glue of *C. grandis* could also originate from either of these two mechanisms. The glue of the *P. philippinicum* eggs, in contrast, is apparently produced by females as a tertiary secretion (extraovarian) [31], mantles the surface of the egg [14,15], and is kept by the **fas** (Figure 4I–P). This glue is probably proteinaceous and includes at least two functional groups, one hydrophilic and oriented towards the substrate and the other hydrophobic and associated with the surface of the egg [14].

The fas themselves, without the glue, do not show substantial adhesive capability. The feet of different animals, both vertebrates and invertebrates, are equipped with fibrillary adhesive hairs as well [7]. These are adhesive because of the compliance of the flexible tips of the adhesive hairs that approach the substrate enough to enable van der Waals interactions with the substrate [7]. The **fas** of both seeds and eggs would, in principle, be able to do so as well, but the **fas** are likely not as flexible and compliant with the substrate.

#### 4.1.2. Adhesive Performance

The main difference between the adhesive performance of the *P. philippinicum* eggs and the C. grandis seeds is actually not the adhesive strength. Although the detachment forces measured reached higher maximum values for the plant seeds (Figure 5B or Figure 7A), the median forces are rather similar, or in some cases even higher for the leaf insect eggs. This is a result of the reliability and efficiency of the adhesion of eggs. The attachment system of the eggs performs consistently in a similar range of forces on different substrates while the detachment forces of seeds revealed strong deviations and a strongly left skewed distribution: most measurements actually resulted in very low forces, but only few measurements of very high forces were obtained. This is likely a result of two aspects of these mechanisms: the properties of the glue and the shape of the **fas**.

The hierarchical splitting and unequal distribution of the pinnae of the eggs of *P. philippinicum* results in a more reliable adaptation to the substrate geometry (Figure 7B). To maximize the attachment force, any adhesive system needs to maximize the actual contact area [7,41]. In comparison to the straight **fas** of the seeds, the pinnae of eggs seem to adapt more efficiently to rough substrates. The same applies to the repetition of the attachment events. Especially on the 12 µm roughness, the detachment forces of the seeds were low. Furthermore, the attachment ratios of the seeds of *C. grandis* were strongly reduced on this roughness (Figure 6D). On the one hand, the diameter of the **fas** of the seeds is approximately in the range of this roughness (Figure 4H), conflicting with proper contact formation. On the other hand, the distribution of the glue makes a difference for both reproductive stages. The glue of the leaf insect eggs forms a dense film, mantling the pinnae and large fractions of the egg surface (Figure 4), while the glue of the plant seeds is mainly distributed on the tips of the fibrils. This potentially results in the higher depth of the surface adaptation and thicker films of glue for the eggs compared to the plant seeds (Figure 7B). Consequently, the eggs make more reliable contact, especially to rough substrates. 

On substrates with different surface chemistry but similar topography, both seeds and eggs showed similarly decreasing detachment forces with an increasing water contact angle. As the surface topography was the same for all three substrates, differences between the two species are the result of the glue properties. The *C. grandis* seeds adhered significantly stronger to hydrophilic substrates. While most plant seed glues are polysaccharides [38,39,42], the glue of the eggs of this particular species of leaf insects has been hypothesized to be a glycoprotein [14,15]. The majority of egg glues in insects are proteinaceous [43,44,45,46,47,48,49] and the amphiphily of the glue properties could well be achieved with glycoproteins, such as in other insect glues [50]. Therefore, a glycoprotein remains a plausible explanation for this particular adhesive system, but the chemical structure of the glue still warrants further investigation. Due to the distance in the phylogenetic relation between *C. grandis* and *P. philippinicum*, it is most likely that these convergently evolved glues in the two species originate from different chemical groups. Comparing the glue of *C. grandis* seeds to other plant seed glues, the forces are similar to the ones reported for cellulose-based mucilage envelopes [38]. However, the basis on which the glue is applied to the substrate is largely different. While the cellulose fibrils and pectines in the known seed glues are anchored in an undisintegrated cell wall, the epidermal cell wall of the *C. grandis* seeds is significantly modified. Nevertheless, the net adhesive forces of other seeds are often much stronger and often more reliable [38], although they do not possess similar specialized **fas** for adaptation to the substrate. The linearity of the seed **fas** causes a less homogenous distribution of the glue film on the substrate compared to the eggs’ pinnae. This interferes with reliable contact formation with the substrate. Therefore, the variance of the detachment forces of the seeds can be higher. If, by chance, a good contact is formed, the detachment forces can be quite high; however, it is also quite likely, due to the unspecialized fibrils, that the contact is unpredictable and can be quite unreliable. The **fas** of *C. grandis* are present from the very beginning as disintegration of the cell wall and spread out after contact with water, whereas the fibers of mucilaginous seeds appear due to their uncoiling from the cell wall after the first hydration [38]. Functionally, the contact formation of both types of seed appendages is similar and the fibrils of both kinds can adapt to the surface profile, but the origin of the fibrils differs. As a result, the size of the cell wall originating **fas** is larger in comparison to the cellulose fibrils of the majority of mucilaginous seeds and might provide less efficient contact. In contrast to cellulose fibrils, the glue generating the adhesion in the seeds examined herein liquefies again with water contact and this can be repeated several times. However, the detachment force decreases over repeating cycles, as the glue is washed off and partly remains on the substrates with every cycle. This effect is stronger in the *C. grandis* seeds as the hierarchically splitting pinnae of the *P. philippinicum* eggs strongly keep the glue on the surface of the egg.

#### 4.1.3. Ecological Differences

Plants and insects are naturally rather different in terms of their demands on their environments. Often, insects are a threat that plants tend to avoid. Interestingly, leaf insects, in particular, visually imitate the leaves of plants to avoid their own predators (spiders, other insects, mammals, birds, and lizards [27,51]). This kind of camouflage evolved quite early in phasmids in general [52,53,54,55,56,57] and leaf insects in particular [58,59]. One result of this type of camouflage is a strong sexual dimorphism in Phylliidae [60] due to the fact that females are sedentary and imitate leaves in the canopy and males need to be mobile to find their mates to reproduce [61]. As a result, the eggs of all phylliids are dropped from the place where the females hide. This results in three aspects of concern, which might require attachment to some kinds of substrates: (1) Eggs dropped to the forest floor might be subjected to flightless parasitic wasps (e.g., Amiseginae), which are often specialized for particular phasmid species [51,62,63]. Attaching the eggs in higher levels of the forest is a widespread strategy to avoid these parasitoids, which evolved independently in many phasmid lineages [64,65]. (2) Localization of the offspring close to the foodplant could guarantee suitable food for the offspring [14,15,66]. (3) Attachment can be used for dispersal [26], as has been shown for many seeds as well [40]. All three scenarios require strong, reliable attachment, as shown for the adhesive system of *P. philippinicum*. Based on the shape of the eggs and their appendages, several other phylliid species likely possess an adhesive capability as well [67], but some seem to have different glue properties and seem to attach better on hydrophobic than hydrophilic substrates (pers. obs). Furthermore, other eggs of several unrelated phasmids also carry glue [26], and some seem to represent similar non-permanent water-responsive adhesive mechanisms [68]. The evolution of such egg surface structures and adhesive systems on eggs is likely a similar complex evolutionary scenario, comparable to other aspects of phasmatodean evolution [69,70], such as the tarsal adhesive systems. These also result from complex environmental conditions and are shaped by interactions with various substrates [7,71,72,73,74,75]. The preferred foodplants that are documented for this insect species are *Mangifera indica* L. (Anacardiaceae), *Nephelium lappaceum* L. (Sapindaceae), and *Psidium guajava* L. (Myrtaceae) and the surface characteristics that are potential adhesive sites are discussed in [15]. Likely, the rough, hydrophilic bark is beneficial for attachment. In contrast to *C. grandis*, the seeds of these plants carry no adhesive capabilities, as far as it has been documented.

For the *C. grandis* seeds, in contrast, it is essential to reach the ground for germination. Therefore, a strong reliable adhesive system is disadvantageous for reproduction. While the eggs can adhere several times, which can be useful for site optimization and to ensure suitable conditions for embryonic development, the *C. grandis* seeds adhere once with noteworthy strength. Presumably, they will be washed off their substrates with the first rain contact and then reach the soil for further germination. The mucilage glue of other plant species is common and studied most in plant species in arid environments or disturbed habitats [38]. Glue-based anchoring in plants has some different advantages compared to that in insects, but some advantages are congruent. Seed glue can be advantageous to sustain proper microenvironments, for example, by retaining humidity or anchoring in a suitable regime [37,76,77,78,79]. This particularly includes anchoring to the ground for germination [80,81]. For plants, which in contrast to insects do not have a moving reproductive stage, dispersal plays a big role and is often facilitated by glues. Long-distance dispersal can, for example, be mediated by migratory animals if the seeds are glued to the feathers of birds or the fur of mammals [79,82,83,84,85]. However, for *C. grandis*, there are no firsthand observations of actual seed dispersal. Nevertheless, mammals and birds are reported to be attracted by fruits and potentially disperse the seeds. These include fruit bats [86,87] and birds [88]. Other larger potential dispersers [89] include humans and elephants [90,91], which feed on the fruits and potentially disperse the seeds via endozoochory. Transport within animals plays a role for some plant seeds [92]. However, for *C. grandis*, the passage of the digestive tract of an animal is not required for successful germination [25] [pers. observation]. Whether the seeds would survive the passage through the digestive system of animals is not known so far [25]. In turn endozoochory is rather unlikely for phasmids in general, and even less likely for leaf insects in particular. It might be possible for the eggs to survive digestion by birds if the gravid female is consumed [93], but feeding experiments of individual eggs to birds exclude the chance of survival of bird digestion for most phasmid eggs [93,94]. In contrast to seeds, insect eggs are, if adhesive, rather designed for specialized tasks and usually adapted to specific attachment sites [17,95,96,97,98,99,100].

### 4.2. Biomimetic Implications

Insects are considered suitable sources for bioinspiration [101] and the same applies for plants [102,103,104]. Both insect egg and plant seed glues studied herein are considered useful templates for water-based glues [14,38,105]. Due to their degradability and potential biocompatibility, they or their derivatives can be potentially directly used for biomedical applications [43]. The differences in the two systems can propagate bioinspiration in two directions. While the adhesive system of the eggs works more reliably for durable long-term adhesion, the seeds adhere stronger but are less reliable. However, the common features of both systems can also provide general considerations for the design of fiber-reinforced glue-based adhesives. Both systems make use of fibrillary structures for glue application and simultaneous structural reinforcement. Fiber reinforcement, in general, can increase the mechanical stability [106,107] and reduce the likelihood of failure of the glue itself (cohesive failure) [108]. Hierarchical splitting of the reinforcing and glue-applying structures can increase the adaption to the substrate roughness [109,110], such as in the leaf insect egg. Unbranching fibers, in contrast, can be used for the short-term initial adhesion but are less useful for reliable long-term adhesion. Hierarchical splitting of these surface structures can increase the contact reliability and reduce the required amount of glue [109,111], whereas the introduction of any fibrillary reinforcements at least increases the stability. Some insect egg proteins are cured by glycosylation [112]. As the system investigated herein works in an enzyme-free environment, it is unlikely that the glue is activated by enzymes but cured by water uptake, e.g., glycation. 

These results might inspire technological applications that reduce the required amount of glue, reducing the material cost or yielding more sustainable adhesive systems. As several other species of Phylliidae and other Phasmatodea carry very different exochorionic structures on their eggs, which are likely involved in adhesion as well, future studies can also further explore the advantages and disadvantages of different modifications of this system in a comparative experimental setting. Natural adhesive systems offer various similar fibrillary adhesive structures that can be informative for biomimetics. These also include temporary adhesive systems such as the hairy adhesive systems of invertebrates or the largely dry fibrous adhesive systems of geckos [7].

## 5. Conclusions

The adhesive systems of the leaf insect *P. philippinicum* and the ivy gourd *C. grandis* consist of similar main components: fibrillary adhesive structures and glue. Both adhesive systems convergently yield strong adhesive forces but perform with different reliability, which correlates with the autecological demands of both reproductive stages. While the **fas** (pinnae) of *P. philippinicum* hierarchically split, the **fas** of *C. grandis* (disintegrated epidermal cells) are more uniform straight fibrils. Both systems facilitate adaption to different surface roughness and perform particularly well on hydrophilic substrates after activation by water contact. While insect eggs attach more reliably in avoidance of parasitoids and foodplant association, the seeds of the ivy gourd are dependent on contact with the soil for germination. Therefore, the strong initial adhesion is usually not repetitive in the ivy gourd. The eggs of the walking leaf, in contrast, are capable of repetitive reattachment over several cycles. Both adhesive systems convergently make use of reinforcing fibers in the glue system and adjust to the surface profile; however, the straight fibers of seeds apparently perform less reliably and are more suitable for initial, temporary attachment while the hierarchically splitting adhesive structures of eggs make more reliable contact and apparently store more glue on the surface. In addition to the choice of a particular morphology for biomimetic applications, the specific requirements can be tuned with different glues in the adhesive system. Nevertheless, both types of glue exemplified by *C. grandis* and *P. philippinicum* are potential candidates for water-soluble biocompatible glues. This study exemplifies the benefits of studying similar mechanisms for a comparison of different perspectives of different but convergently evolved systems for biomimetics. The specific requirements result in different modifications of similar mechanisms for the respective tasks and enable an assessment of the underlying constraints. Examination of further similar adhesive mechanisms on the eggs of other walking leaf species, other phasmid species in general, and the seeds of further plant species with similar fibrillary adhesive systems might yield more insights into the different modifications, which can represent an informative toolbox for biomimetics. 

## Data Availability

The raw data of the pull-off experiments of both the *Phyllium philippinicum* eggs and the *Coccinia grandis* seeds from substrates with different surface roughness and surface chemistry are made available via the Supplementary Materials.

## Supplementary Materials

The following supporting information can be downloaded at: https://www.mdpi.com/article/10.3390/biomimetics7040173/s1, Excel Sheet S1: force measurements.
